# Body Weight Loss and Tissue Wasting in Late Middle-Aged Mice on Slightly Imbalanced Essential/Non-essential Amino Acids Diet

**DOI:** 10.3389/fmed.2018.00136

**Published:** 2018-05-17

**Authors:** Giovanni Corsetti, Evasio Pasini, Claudia Romano, Riccardo Calvani, Anna Picca, Emanuele Marzetti, Vincenzo Flati, Francesco S. Dioguardi

**Affiliations:** ^1^Division of Human Anatomy and Physiopathology, Department of Clinical and Experimental Sciences, University of Brescia, Brescia, Italy; ^2^Cardiac Rehabilitation Division, IRCCS Istituti Clinici Scientifici Maugeri (ICS Maugeri), Lumezzane, Italy; ^3^Department of Geriatrics, Neurosciences and Orthopedics, Catholic University of the Sacred Heart, Rome, Italy; ^4^Department of Biotechnological and Applied Clinical Sciences, University of L'Aquila, L'Aquila, Italy; ^5^Department of Clinical Sciences and Community Health, University of Milan, Milan, Italy

**Keywords:** aging, nutrition, diet, muscle atrophy, body wasting, food choice

## Abstract

**Objective:** Inadequate protein intake can impair protein balance thus leading to skeletal muscle atrophy, impaired body growth, and functional decline. Foods provide both non-essential (NEAAs) and essential amino acids (EAAs) that may convey different metabolic stimuli to specific organs and tissues. In this study, we sought to evaluate the impact of six diets, with various EAA/NEAA blends, on body composition and the risk of developing tissue wasting in late middle-aged male mice.

**Methods:** Six groups of late middle-aged male mice were fed for 35 days with iso-nutrients, iso-caloric, and iso-nitrogenous special diets containing different EAA/NEAA ratios ranging from 100/0% to 0/100%. One group fed with standard laboratory rodent diet (StD) served as control. Preliminarily, we verified the palatability of the diets by recording the mice preference, and by making accessible all diets simultaneously, in comparison to StD. Body weight, food and water consumption were measured every 3 days. Blood and urine samples, as well as heart, kidneys, liver, spleen, triceps surae, retroperitoneal WAT, and BAT were harvested and weighed.

**Results:** Mice consuming NEAA-based diets, although showing increased food and calorie intake, suffered the most severe weight loss. Interestingly, the diet containing a EAA/NEAA-imbalance, with moderate NEAAs prevalence, was able to induce catabolic stimuli, generalized body wasting, and systemic metabolic alterations comparable to those observed with diet containing NEAA alone. In addition, complete depletion of retroperitoneal white adipose tissue and a severe loss (>75%) of brown adipose tissue were observed together with muscle wasting. Conversely, EAA-containing diets induced significant decreases in body weight by reducing primarily fat reserves, but at the same time they improved the clinical parameters. On these basis we can deduce that tissue wasting was caused by altered AA quality, independent of reduced nitrogen or caloric intake.

**Conclusion:** Our results indicate that diets containing an optimized balance of AA composition is necessary for preserving overall body energy status. These findings are particularly relevant in the context of aging and may be exploited for contrasting its negative correlates, including body wasting.

## Introduction

Skeletal muscle contractile proteins represent a ready to use amino acids reservoir during fasting or stress. Therefore, inadequate protein intake can easily impair protein balance and leads to skeletal muscle atrophy, impaired muscle growth, and functional decline (1).

Dietary proteins provide Nitrogen (N)-rich amino acids (AAs) that are pivotal to maintain cell integrity in mammals. From a nutritional point of view, AAs can be classified as either non-essential (NEAAs), or essential (EAAs) depending on the possibility to be synthesized endogenously or not (2).

We recently showed that aged mice on standard diet (StD) supplemented with a balanced EAAs formulation had increased lifespan, improved mitochondrial biogenesis and morphological and molecular changes in heart, skeletal muscle, and adipose tissue (3–6). The protective role of an EAA-rich diet was also observed in the liver of rats with chronic ethanol consumption (7, 8), in the kidneys of rosuvastatin-treated mice (9), and during wound healing (10). Furthermore, *in vitro* data showed that variations in the EAA/NEAA ratio regulated cancer cell survival or death (11). These data suggest that varying dietary concentrations of EAA/NEAA may affect the whole-body metabolism. However, the impact of different EAA/NEAA ratios on body growth and tissue homeostasis is not yet fully appreciated.

Adequate calorie intake is crucial for growth and lifespan. However, even sufficient caloric intake but with a defective nitrogen (N) supply can induce “*under-nutrition*” (12). Dietary proteins contain an excess of NEAAs (EAA/NEAA < 1, with a % ratio of 45/55, respectively) (13). We therefore investigated the chronic effects of six iso-caloric and iso-nitrogenous diets with specific stoichiometric mixtures of free AAs varying in EAA/NEAA ratios as exclusive N source, compared with a standard laboratory rodent chow (StD), in late middle-aged male mice. We measured relevant clinical parameters such as body weight and length, organ weight, food and water consumption, and standard urinary and circulating metabolic markers. Finally, we evaluated the mice dietary preference among all of the available food options to assess the impact of various EAA/NEAA ratios on palatability.

## Materials and methods

Nine-month-old male Balb/C mice purchased from Envigo (Horst, The Netherlands) were individually housed in plastic cages with white wood chips for bedding, in a quiet room under controlled lighting (12h day/night cycle) and temperature (22 ± 1°C) conditions. Mice had *ad libitum* access to water and food. Body weight as well as food and water consumption were measured every 3 days. Food intake was recorded and cages were inspected for food drop and/or fragments of wasted pellets. Animals were regularly evaluated by veterinary physicians for their health and maintenance of normal daily and nocturnal activities and for criteria of increased disease burden according to ethics standards for animal studies. The study was conducted in accordance with the Italian Ministry of Health and complied with the “*The National Animal Protection Guidelines*.” The Ethics Committee for animal experiments of the University of Brescia (OPBA, *Organismo Preposto per il Benessere Animale* = *Organism Controlling Animal Wellbeing*) and the Italian Ministry of Health (DGSAFV, Direzione Generale della Sanità Animale e dei Farmaci Veterinari authorization n. 458/2015) approved all of the experimental procedures.

### Diet composition

We developed six specific diets containing different EAA/NEAA ratios ranging from 100/0% to 0/100%:

1. The EAA-exclusive diet (EAA-Ex) contained EAAs (100%) as the unique source of N, in a formulation tailored to mammal needs (14) and widely applied in clinical and experimental studies (3, 5, 7, 9, 10, 15).
2. The EAA-rich diet (EAA-R) contained a mixture of EAAs (~84%) and NEAAs (~16%).
3. The casein-like diet (Cas-AA) contained free AAs equivalent to casein composition (~49% EAAs and ~51% NEAAs) as exclusive N source.
4. The casein protein diet (Cas-Prot) contained casein protein as the exclusive source of N.
5. The NEAA-rich diet (NEAA-R) contained a mixture of EAAs (~33%) and NEAAs (~67%).
6. The NEAA-exclusive (NEAA-Ex) diet contained only free NEAAs (100%) as exclusive N source, formulated on the basis of the NEAA content in casein.

A standard laboratory rodent chow (StD) (*Mucedola srl*, Milan, Italy) was used as the reference diet with a N source represented by vegetal and animal (fish) proteins.

All special diets were iso-nutrients, iso-caloric, and iso-nitrogenous and were expressly prepared for *Nutriresearch s.r.l*. (Milan, Italy) by Dottori Piccioni (Milan, Italy) in accordance with AIN76-A/NIH-7 rules. The composition of each diet is summarized in Table 1.

**Table 1:** Diet composition.

	**EAA-Ex**	**EAA-R**	**Cas-AA**	**Cas-Prot**	**StD**	**NEAA-R**	**NEAA-Ex**
KCal/Kg	3995	3995	3995	3995	3952	3995	3995
Carbohydrates %	61.76	61.76	61.76	61.76	54.61	61.76	61.76
Lipids %	6.12	6.12	6.12	6.12	7.5	6.12	6.12
Nitrogen%	20^*^	20^*^	20^*^	20^∧^	21.8°	20^*^	20^*^
Proteins: % of total nitrogen content	0	0	0	100	95.93	0	0
Free AA: % of total nitrogen content	100	100	100	0	4.07	100	100
EAA/NEAA (% in grams)	100/0	84/16	49/51		–	33/67	0/100
**FREE AA COMPOSITION (%)**							
L-Leucine *(bcaa)*	31.25	13.53	9.5	–	–	9.4	–
L-Isoleucine *(bcaa)*	15.62	9.65	6	–	–	4.7	–
L-Valine *(bcaa)*	15.62	9.65	6.5	–	–	4.7	–
L-Lysine	16.25	11.60	7	–	0.97	6.24	–
L-Threonine	8.75	8.70	4	–	–	2.7	–
L-Hystidine	3.75	11.60	2.8	–	–	1.1	–
L-Phenylalanine	2.5	7.73	5	–	–	0.8	–
L-Cysteine	–	–	0.8	–	–	–	–
L-Cystine	3.75	6.54	–	–	0.39	1.1	–
L-Methionine	1.25	4.35	2.5	–	0.45	0.4	–
L-Tyrosine	0.75	5.80	5	–	–	2.6	1.0
L-Triptophan	0.5	3.38	1.3	–	0.28	0.01	–
L-Alanine	–	–	3.2	–	–	24.0	35.0
L-Glycine	–	–	2.4	–	0.88	10.39	15.0
L- Arginine	–	–	3.4	–	1.1	13.5	14.0
L-Proline	–	–	9.5	–	–	8.2	12.0
L-Glutamine	–	–	9.5	–	–	3.0	12.0
L-Serine	–	1.95	5.1	–	–	4.1	*6.0*
L-Glutamic Acid	–	–	9.5	–	–	2.5	2.0
L-Asparagine	–	–	3.5	–	–	0.79	2.0
L-Aspartic Acid	–	–	3.5	–	–	1.1	1.0
Ornitine-αKG	–	1.94	–	–	–	–	–
N-acetyl-cysteine	–	0.78	–	–	–	–	–

* *Nitrogen (%) from free AA only*.

° Nitrogen (%) from vegetable and animal proteins and added AA;

∧ *whole casein protein; EAA-Ex, essential amino acids exclusively; EAA-R, EAA-Rich diet; Cas-AA, Casein like AA exclusively; Cas-Prot, Casein protein exclusively; StD, Standard diet; NEAA-R, non-essential amino acids rich; NEAA-Ex, non-essential amino acids exclusively. The gray column represents the standard reference diet. The black line represents the limit between EAAs (upside) and NEAAs (beneath). bcaa, branched chain amino-acids*.

To exclude the possibility of growth failure due to underfeeding (16), we verified the palatability of the special diets, in comparison to the StD, by recording the rodent choice among all these diets available simultaneously to the mice.

### Food preference test (mixed diets)

Six tests of food choice, each with three diets mixed in various blends, were carried out to identify patterns of preference/avoidance of specific diets. Four animals for each test had access to an equal amount of three diets. These diets, separated from each other, were presented simultaneously to the animals in a manger for about 4 weeks, so that the mice could freely choose their preferred pellets. The position of each diet in the manger was swapped regularly to prevent a habit of eating the same diet from a fixed position. Once we had observed that none of the special diets were rejected, we proceeded to the feeding protocol to assess the effects of each individual diet.

### Metabolic effects of single diets

Animals were randomly assigned to seven groups. Each group was fed for 35 days exclusively with a specific diet, i.e., EAA-Ex diet (*n* = 10), EAA-R diet (*n* = 10), Cas-AA diet (*n* = 10), Cas-Prot (*n* = 10), StD (*n* = 10), NEAA-R diet (*n* = 20), NEAA-Ex diet (*n* = 20). Food and water were accessible *ad libitum*.

### Sample collections

Animals were sacrificed by cervical dislocation at the end of the treatment (day 35) or earlier according to ethical criteria of increased disease burden, mostly based on weight loss. Nose-tail length (body length) and glycaemia from tail blood were measured in all animals. Blood samples from the heart as well as urine were collected upon sacrifice. Subsequently, heart, kidneys, liver, spleen, *triceps surae*, retroperitoneal white adipose tissue (rpWAT), and brown adipose tissue (BAT) were quickly harvested and weighed.

### Blood and urine analysis

Analyses were carried out at the “*Division of Laboratory Animals*” of the “*National Reference Centre for Animal Welfare*” (IZSLER-Bs, http://www.izsler.it). Blood cell count was assessed with a Cell-Dyn 3700 laser-impedence cell counter (Abbott Diagnostics Division, Abbott Laboratories, Lake Bluff, IL). Albumin and creatinine concentrations were measured in serum and urine using a biochemical automatic analyzer ILab Aries (Instrumentation Laboratory, Bedford, MA). Neutrophils to Lymphocytes ratio (NLR) was determined as a marker of inflammation (17, 18).

### Statistics

Data are expressed as means ± standard deviation (SD). Comparisons among dietary groups were performed by one-way ANOVA followed by Bonferroni *post-hoc* test, as appropriate. Statistical significance was set at *p* < 0.05.

## Results

### Food preference test (mixed diets)

**Figure 1:**
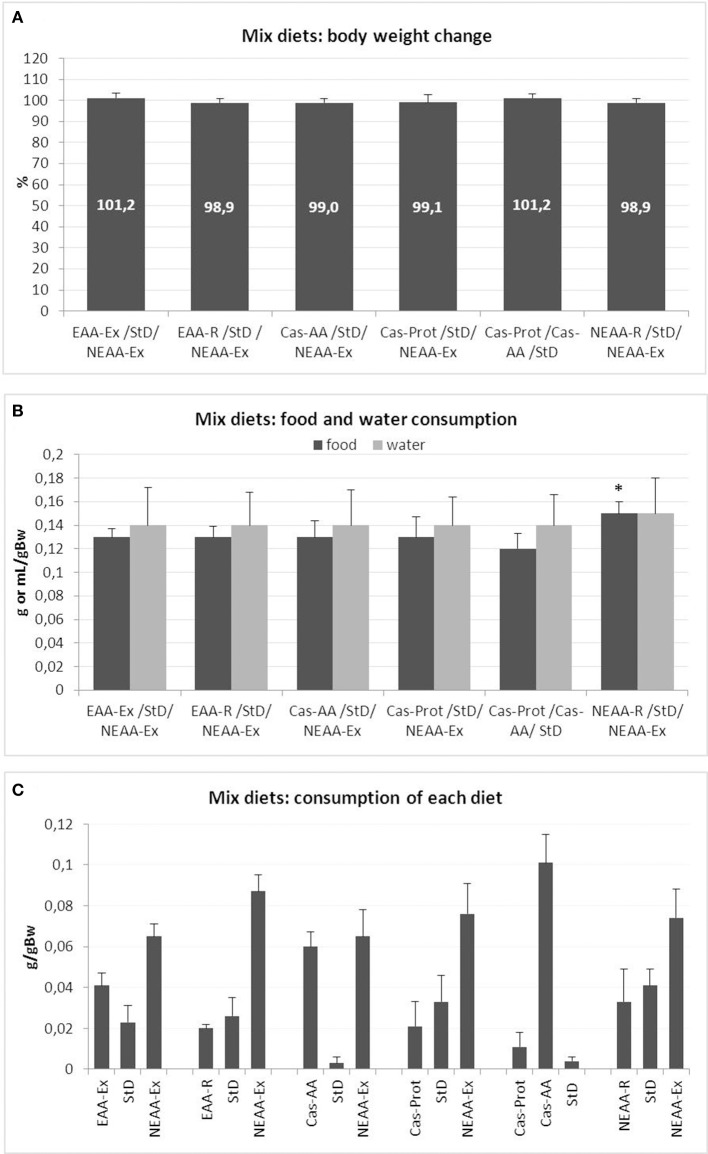
**(A–C)** Feeding protocol 1 (Mix diets preference choice): comparison of weight change from baseline, food and water intake and feeding preference at free access to several diets in various combinations. **(A)** Mean change of body weight from baseline (in % ± *sd*) to the end of feeding with mix diets. Body weight at day 0 = 100%. None of the diet mixes, even if some had a heavily imbalanced EAA/NEAA ratio, modified body weight. **(B)** Mean daily food (g/gBW, *F* = 2.81) and water (mL/gBW, *F* = 0.08) consumption during mixed diet preference choice testing (mean ± *sd*). Only in the diet combination StD/NEAA-R/NEAA-Ex, there was a small but significant increase in total daily food consumption (*post-hoc* test: ^*^p < 0.05 vs. Cas-Prot/Cas-AA/StD. **(C)** Feeding preference of individual diets at free access as assessed by daily food intake (*g/gBW*). In all diet combinations, the most consumed food was the NEAA-Ex diet and when present, the Cas-AA diet.

The mice body weight did not change significantly during any of the food preference tests (Figure 1A). Similarly, no significant differences were observed in water consumption (Figure 1B) or food intake, with each animal consuming about 0.13 g/g bw^−1^ of food (about 3.8 g/day, for a total 15.2 Kcal/day) (Figure 1B). Among all the available dietary choices, the NEAA-Ex resulted the preferred option (Figure 1C). A significant increase in total daily food intake was also observed for NEAA-R diet and NEAA-Ex diets in combination with StD (StD/NEAA-R/NEAA-Ex). The Cas-AA diet was the only other option that was well appreciated, almost as much as the NEAA-Ex diet (Cas-AA/StD/NEAA-Ex), even when no NEAA-Ex diet was presented to the animals (Cas-Prot/Cas-AA/StD). Conversely, Cas-Prot diet was not a preferred option in any of the combination presented to the animals (Cas-Prot/StD/NEAA-Ex and Cas-Prot/Cas-AA/StD). When animals could choose between Cas-Prot and Cas-AA diets, they almost exclusively opted for the Cas-AA diet (Cas-Prot/Cas-AA/StD) (Figure 1C).

### Metabolic effects of single diets

#### Phenotypical modifications, body weight, food, and water consumption

##### Animal growth

Data recorded from animals fed Cas-AA and Cas-Prot diets did not differ significantly from those on StD. For this reason, we have reported in tables all the analytical data relative to StD only, in order to simplify reading.

**Figure 2:**
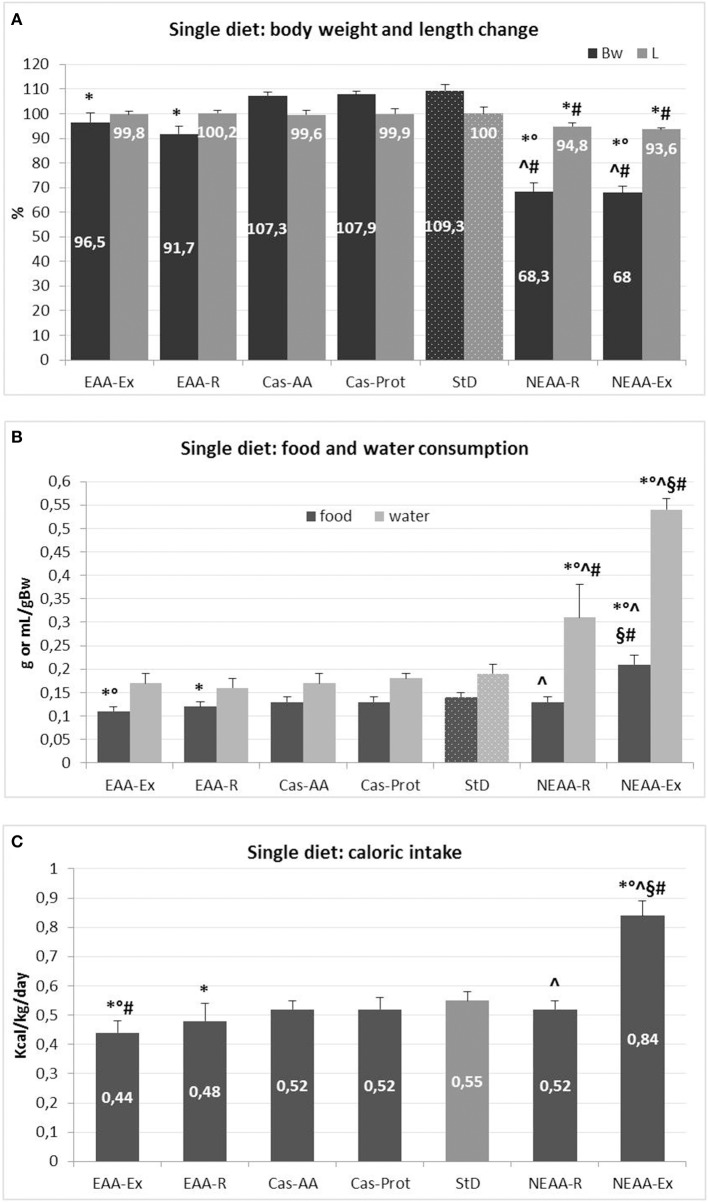
**(A–C)** Feeding protocol 2 (Metabolic effects of individual diets exclusively presented to animals for 35 days or 21 days for NEAA-Ex and NEAA-R diets). **(A)** Mean change (% ± *sd*) of body weight and body length from baseline for each diet (at day 0 = 100%). Dotted columns represent the reference diet. **(B)** Mean daily food (g/gBW) and water (mL/gBW) intake (± *sd*). Dotted columns represent the reference values. **(C)** Mean daily caloric intake (Kcal/gBW ± *sd*). The gray column represents the reference diet. *Post-hoc* test: ^*^p < 0.05 vs. StD, °*p* < 0.05 vs. EAA-R, ^#^vs. Cas-AA/Cas-Prot, ^∧^*p* < 0.05 vs. EAA-Ex, ^§^*p* < 0.05 vs. NEAA-R.

Mean changes in body weight and length for each diet are expressed as % from baseline (beginning of the treatment, time 0 = 100%) (Figure 2A). Body weight of StD-fed animals increased by ~9%, whereas animals fed Cas-AA and Cas-Prot diets showed body weight increase of about 7%. Instead, a decrease of 3.5% and 8% (*p* < 0.05) was found in animals fed EAA-Ex and EAA-R, respectively, although the body length did not change when compared with control diets (Cas-AA, Cas-Prot and StD). A more pronounced and progressive weight loss was found in animals fed NEAA-R and NEAA-Ex diets. Because suffering from increased disease burden, these animals were frequently monitored by veterinarians. As shown in Figure 2A, after 21 days of NEAA-R and NEAA-Ex dietary regimens, these mice lost about 30% of their initial body weight and showed a significant reduction in body length (about 5 and 6.5%, respectively).

Besides dramatic weight loss, no other signs of suffering, such as hypothermia, apathy, persisting staggering, bleeding, persisting diarrhea, labored breathing, cyanosis, complete lack of food intake or dehydration (19), were reported. However, to avoid further discomfort and in accordance with ethical standards, all animals belonging to these two feeding groups were euthanized by cervical dislocation. None of the animals following other dietary regimens died prematurely or had to be prematurely sacrificed. Following the experimental protocol, they were euthanized after 35 days.

##### Food and caloric intake

Mean daily food and caloric intake for each diet are summarized in Figures 2B,C. The NEAA-Ex fed animals had higher food intake and, hence, higher caloric intake when compared with StD-fed mice or those on any of the other special regimen. Both EAA-Ex fed and EAA-R fed animals had the lowest food intake (−21 and −14%, respectively) and therefore lower caloric intake, compared with mice fed StD.

##### Water intake

Mean daily water consumption for each diet is summarized in Figure 2B. Water intake was similar across EAAs-based and Cas-derived diets, but it was significantly higher in both NEAAs-based diets. Water consumption markedly increased by 160% in the NEAA-R fed group and up to about 280% in the NEAA-Ex fed group compared with StD-fed mice.

#### Blood and urine analyses of metabolic markers

Several metabolic markers were measured across dietary groups and results are reported in Table 2. Blood levels of glucose, albumin, and creatinine were significantly lower in both NEAAs-fed animals compared with all other groups. Conversely, EAA-Ex and EAA-R fed mice had the highest plasma albumin values.

**Table 2:** Blood and urine parameters (mean ± *sd*) at the end of treatment.

	**EAA-Ex**	**EAA-R**	**StD**	**NEAA-R**	**NEAA-Ex**	***F***	***p***
**BLOOD**							
Glucose *(mg/dL)*	133.2 ± 2.63	135.6 ± 2.3	128.4 ± 12.61	97.3 ± 8.62^*^°^∧^	109.8 ± 6.64^*^°^∧^	47.64	0.000
Hemoglobin *(g/dL)*	15.1 ± 0.41^*^	15.9 ± 0.62^*^	14,1 ± 1.64	15.3 ± 0.34^*^°	13.2 ± 0.64^*^°^∧^^§^	15.16	0.000
NLR	0.19 ± 0.11^*^	0.26 ± 0.16^*^	0.35 ± *0.07*	1.79 ± 0.76^*^°^∧^	1.67 ± 0.24^*^°^∧^	47.76	0.000
Albumin *(g/L)*	28.68 ± 0.75^*^	30.64 ± 2.35^*^	26.57 ± 1.97	24.75 ± 1.56^*^°^∧^	22.6 ± 1.68^*^°^∧^^§^	32.90	0.000
Creatinine *(μmol/L)*	46.98 ± 2.26	46.06 ± 3.96	44.95 ± 7.9	44,6 ± 2.3	35,92 ± 4.5^*^°^∧^^§^	9.11	0.000
**URINE**							
Albumin *(g/L)*	2.35 ± 0.35	2.22 ± 0.34	2.66 ± 0.23	0.6 ± 0.5^*^°^∧^	0.7 ± 0.3^*^°^∧^	31.33	0.000
Creatinine *(μmol/L)*	4502 ± 244	5455 ± 1350	4947 ± 344	6147 ± 958^*^°^∧^	8842 ± 591^*^^°^^§^^∧^	22.47	0.000

Standard reference diet is shown in the gray column. Post-hoc test:

* *p < 0.05 vs. StD*,

° *p < 0.05 vs. EAA-R*,

∧ *p < 0.05 vs. EAA-Ex*,

§ *p < 0.05 vs. NEAA-R*.

NEAA-Ex fed animals showed reduced hemoglobin levels, whereas a 5-fold higher NLR was found in animals fed with either NEAA-based diets compared with StD-fed counterparts. Higher NLR indicates increased inflammatory activity in NEAA-based diets fed - but otherwise healthy - animals. Conversely, NLR was lower with EAAs-based diets, suggesting an anti-inflammatory effect of those diets. In mice on NEAA-based diets, urinary albumin levels were substantially reduced (about −75%), while creatinine excretion levels were increased by 24 and 79% in NEAA-R and NEAA-Ex fed animals, respectively.

#### Organ weight

Organs from any experimental group did not show macroscopic alterations. Organ weight decreased markedly in NEAA-R and NEAA-Ex diets compared with other dietary regimens. Notably, rpWAT was lost in these animals. Mild reductions of organ weight were also observed in EAA-based diets (Table 3).

**Table 3:** Mean body weight (*g*), body length (*cm*), and organ weight (*g*) at the end of treatment.

	**EAA-Ex *n = 10***	**EAA-R *n = 10***	**StD *n = 10***	**NEAA-R *n = 20***	**NEAA-Ex *n = 20***	***F***	***p***
Body weight	28 ± 1.22^*^	27.6 ± 1.14^*^	29.67 ± 1.97	17.83 ± 1.17^*^°^∧^	17.5 ± 0.9^*^°^∧^	*199.7*	*0.000*
Body length	10,06 ± 0.13	10,1 ± 0.11	10,08 ± 0.28	9,56 ± 0.15^*^°^∧^	9,43 ± 0.08^*^°^§^	*64.00*	*0.000*
Heart	0.18 ± 0.01	0.18 ± 0.01	0.20 ± 0.06	0.13 ± 0.01^*^°^∧^	0.15 ± 0.02^*^	*8.95*	*0.000*
Kidneys	0.57 ± 0.05	0.58 ± 0.01	0.58 ± 0.06	0.30 ± 0.04^*^°^∧^	0.32 ± 0.03^*^°^∧^	*122.99*	*0.000*
Liver	1.43 ± 0.03^*^	1.47 ± 0.13^*^	1.68 ± 0.2	0.65 ± 0.07^*^°^∧^	0.78 ± 0.23^*^°^∧^	*90.30*	*0.000*
Spleen	0.09 ± 0.01^*^	0.10 ± 0.01^*^	0.15 ± 0.04	0.05 ± 0.01^*^°^∧^	0.05 ± 0.01^*^°^∧^	*43.0*	*0.000*
rpWAT	0.18 ± 0.03^*^	0.10 ± 0.04^*^^∧^	0.21 ± 0.02	0	0	*33.45*	*0.000*
BAT	0.16 ± 0.03	0.12 ± 0.01^*^^∧^	0.17 ± 0.01	0.06 ± 0.01^*^°^∧^	0.04 ± 0.009^*^°^§^	*130.77*	*0.000*
Triceps surae	0.21 ± 0.01^*^	0.22 ± 0.03^*^	0.26 ± 0.02	0.08 ± 0.02^*^°^∧^	0.07 ± 0.01^*^°^∧^	*199.21*	*0.000*

rpWAT, retroperitoneal white adipose tissue; BAT, brown adipose tissue. Standard reference diet is shown in the gray column. Post-hoc test:

* *p < 0.05 vs. StD*,

° *p < 0.05 vs. EAA-R*,

∧ *p < 0.05 vs. EAA-Ex*,

§ *p < 0.05 vs. NEAA-R*.

Organ weight normalized to body weight was calculated and results are reported in Table 4. While relative organ weights were similar in EAA-based fed mice compared with StD-fed animals, significant reductions in relative weights of all organs, except for the heart, were observed with NEAA-based feeding. WAT tissue was absent in animals fed with NEAA-R and NEAA-Ex diets. Conversely, relative heart weight was higher in the NEAA-Ex fed group compared with all other dietary regimen. Similarly, NEAA-R fed animals showed a trend toward increased heart weight. However, it is worth mentioning that the body weight of these animals decreased markedly. Hence, despite showing absolute tissue loss (Table 3), the heart suffered reduced wasting compared to other organs and tissues.

**Table 4:** Organ weight normalized to body weight (%) at the end of treatment.

	**EAA-Ex *n = 10***	**EAA-R *n = 10***	**StD *n = 10***	**NEAA-R *n = 20***	**NEAA-Ex *n = 20***	***F***	***p***
Heart	0.63 ± 0.02	0.67 ± 0.04	0.66 ± 0.17	0.74 ± 0.05	0.86 ± 0.13^*^°^∧^^§^	23.65	0.000
Kidneys	2.04 ± 0.15	2.12 ± 0.08^*^	1.93 ± 0.13	1.68 ± 0.12 ^*^°^^∧^^	1.76 ± 0.18 ^*^°^^∧^^	18.35	0.000
Liver	5.11 ± 0.24	5.37 ± 0.42	5.6 ± 0.4	3.66 ± 0.25^*^°^^∧^^	4.33 ± 1.4^*^°^^∧^^	9.23	0.000
Spleen	0.34 ± 0.05	0.35 ± 0.04	0.39 ± 0.13	0.26 ± 0.03^*^°^^∧^^	0.26 ± 0.05^*^°^^∧^^	9.22	0.000
rpWAT	0.64 ± 0.12	0.37 ± 0.13^*^^^∧^^	0.72 ± 0.07	0	0	27.87	0.000
BAT	0.56 ± 0.09	0.45 ± 0.05^*^^^∧^^	0.51 ± 0.08	0.31 ± 0.05^*^°^^∧^^	0.23 ± 0.04^*^°^∧^^^§^^	69.51	0.000
Triceps surae	0.78 ± 0.06	0.80 ± 0.05	0.82 ± 0.06	0.45 ± 0.03^*^	0.41 ± 0.05^*^°^^∧^^	158.28	0.000

Standard reference diet is shown in the gray column. Post-hoc test:

* *p < 0.05 vs. StD*,

° *p < 0.05 vs. EAA-R*,

∧ *p < 0.05 vs. EAA-Ex*,

§ *p < 0.05 vs. NEAA-R*.

## Discussion

Several studies have shown the influence of varying dietary concentrations of EAA/NEAA ratio on whole-body metabolism. However no data are available on their effects on body growth and wasting. The present study was therefore undertaken to provide insights on this piece of information. Our results suggest that differential AA composition of food (although iso-caloric and iso-nitrogenous) has substantial impact on body and organ weight of healthy animals. In particular, tissue wasting, changes in metabolic markers and relevant effects on activation of inflammation seem to be the most affected processes. Indeed, alteration of EAA/NEAA standard ratio even with slight reduction of EAAs, leads to severe catabolic imbalance and the development of body wasting and premature death. Interestingly, changes observed in body weight did not depend solely upon caloric intake, but were also linked with the ratio EAA/NEAA supplied by diets.

EAA-based diets (EAA-Ex and EAA-R) were consumed in significantly lower quantities than StD thus leading to a decreasing proportion of caloric intake. Previous studies have indicated an association between increased plasma AA concentration and decreased appetite. Indeed, the elevated plasma concentrations of EAAs can trigger satiety signals, thereby decreasing food intake (20). However, in spite of lower consumption of EAAs-based diets, no reductions in body weight were observed. This suggests that the limited calorie intake might not be the only condition accounting for weight loss when comparing the quality of AAs ingested. Possibly, the low amount of EAA-based food consumed is sufficient to induce satiety and to provide a metabolically active N source in those animals.

On the other hand, NEAA-R diets were consumed in the same amount of control diets whereas NEAA-Ex diets were consumed in excess respect to StD. However, both NEAA-based diets induced dramatic morphological and clinical changes in animals already after 3 weeks of feeding. The substantial decrease in organ weight and body length and the heavy clinical changes following NEAA-Ex diets were in line with previous findings reporting the deleterious effect of EAA deficiency (21).

More interestingly, a similar damage was induced in the same time frame by slight excesses of NEAAs. Furthermore, an important new observation is that higher NEAA-based diets intake dramatically increased water consumption. This finding could be explained by substantial muscle proteolysis (as indicated by relevant urinary creatinine) and concomitant hyperosmolarity due to the release of different products of N catabolism, and not only AAs (including EAAs) into the bloodstream. While animals fed EAA-Ex diet slightly decreased rpWAT, those on EAA-R diet lost a much higher amount of rpWAT, whereas other clinical parameters did not vary if compared with the control groups. This suggests that dietary EAA composition plays an important role in adipocyte metabolism. Our data are in line with a previous study showing the effects of EAAs supplementation on longevity as associated with improvements in mitochondria function and replication (5).

Notably, both NEAA-based diets induced complete loss of rpWAT with simultaneous decrease of BAT, although calorie intake was significantly higher than in StD-fed animals. WAT is the most plastic organ within the body, able to store and release lipids in response to changes in calorie intake and energy needs. Therefore, rapid fat loss suggests a possible change in the balance of substrates used for energy production and/or increased energy expenditure. Compared with the NEAA-Ex diet alone, this finding confirms previous observations showing that mice maintained on an L-leucine-deficient diet for 7 days show reduced fat mass and lipogenic activity, enhanced energy expenditure and lipolysis in WAT and increased thermogenesis in BAT (22). However, the NEAA-R diet contained the same amount of L-leucine as the Cas-AA diet, so other factors are likely to be involved in fat tissue loss. It is possible that, the modest alteration of EAA/NEAA ratio played a more critical role than L-leucine concentration *per se* in directing WAT cell metabolism. In light of a previous work showing that supplementation with a balanced EAAs mixture extends lifespan, mitochondriogenesis and mitochondrial function (5), the lack of EAAs, or even an excess of NEAAs, might blunt or impair mitochondrial integrity and function.

Another relevant observation is that EAA-based diets increased serum albumin concentration and reduced inflammation, as indicated by lower NLR values, compared with controls and NEAA-based diets. This may indicate that an excess of EAAs induces an anti-inflammatory response. This is in line with our previous studies showing that dietary supplementation with balanced EAAs mixtures reduced oxidative stress, thus slowing cellular aging (3, 5, 7, 9).

Our data also showed that malnutrition induced by NEAA-Ex diet led to a rapid decrease of circulating proteins such as hemoglobin and albumin, whereas the NEAA-R diet decreased only albumin. This is in line with what occurs in undernourished patients (23, 24), and is probably due to reduced protein synthesis and turnover dependent on poor EAA availability. Hypoalbuminemia cannot be explained by loss of albumin through the kidney, as our data showed that albuminuria decreased in NEAA-based diet fed animals. This rather suggests that the kidney in these animals works properly and tries to counterbalance hypoalbuminemia by reducing renal albumin loss. In our healthy animals, “nitrogen quality” of NEAA-based diets caused a state of chronic inflammation, highlighted by substantial NLR increase. Inflammatory activation, in turn, may contribute to a hypercatabolic state, which can further aggravate hypoalbuminemia. This would suggest that in any case, where hypoalbuminemia and inflammation are contemporarily detected, N malnutrition should be suspected and, if confirmed, treated by primarily supplying adequate amounts of EAAs.

Another relevant finding of our study is the increased mortality (within 3 weeks) of animals fed both NEAA-based diets. These animals were euthanized for ethical reasons though they had no signs of suffering. However, the progressive dramatic loss of body weight of over 30% was indicative of poor prognosis in the short term (19). Loss of muscle and fat mass is a common feature of body wasting conditions (25). The underlying mechanism of muscle loss is most probably an impaired balance between protein synthesis and degradation (26). It is not surprising that the NEAA-Ex diet led to rapid weight loss and death, as it does not contain any EAAs. However, even a modest increase in NEAAs (as in NEAA-R diet) induced a very similar significant wasting.

### NEAA enriched diets are preferred by the animals although can prove deadly

NEAA-Ex diet was always preferentially consumed by the animals, both in the preference test and as an individual diet, despite its detrimental effects. This partially contradicts previous works (the first from the 1930s) showing that animals fail to feed and grow, when given an EAA-deficient (or imbalanced) diet. It was therefore concluded that they would not eat an unfavorable diet in order to avoid “useless” or potentially harmful food intake (16, 27). Recently, several studies investigated the neural and molecular mechanisms underlying the choice of food based on EAA availability, but results have often been contradictory (28, 29). Our experimental evidence based on free access to diets containing varying EAA/NEAA ratios, clearly demonstrated that animals did not reject unbalanced NEAA-based diets, even for a prolonged time. It can therefore be concluded that the criterion of food choice is not dependent on the “quality” of AAs mixture.

These findings may have important practical consequences. Indeed, the prevailing perspective is that the behavior test of diet rejection/preference in laboratory animals is a useful nutritional tool to evaluate protein quality and optimal EAA balance of diet (16). Though, our data contradict this proposition. The differences may depend on animal species (mice vs. rats). Indeed, although mice and rats respond quite similarly to AAs, small differences in preferences for some individual AAs have been reported (30). In mice, the taste plays a pivotal role in food choice. For instance, some AAs are avoided because they are perceived as bitter, while other AAs, mostly NEAAs, are more palatable because perceived as sweet or *umami* tasting: these behavioral thresholds depend heavily on AA concentrations (30). This could explain why our NEAA-Ex diet was preferred among all other diets.

### Clinical implications

AAs have multiple metabolic roles besides serving as substrates for protein synthesis, which is quite independent of caloric intake. Indeed, optimized EAA stoichiometric ratios positively affect satiety and anabolic regulations by controlling the insulin-mTOR signaling pathway as previously suggested (4, 31). Nowadays, in human clinical settings, ovalbumin is still the reference protein for estimating nutritional status despite containing an EAA/NEAA ratios < 1 (32). Our data suggest that this ratio might represent a borderline EAA/NEAA balance, only slightly supportive of life in healthy subjects. Therefore even relatively small decreases in EAAs or increases of NEAAs could rapidly cause tissue and organ wasting. This is particularly relevant in older populations considering that food intake undergoes substantial changes over the life course implying variations in body energy fuel reservoirs (33). Indeed, the preservation of overall bodily energy status is critical for successful aging and a breach in the nutritional status makes older adults more vulnerable to internal and/or external stressors, leading them to a condition referred to as “*anorexia of aging*” (33).

Body wasting, poor endurance, reduced physical performance, slow gait speed, and impaired mobility represent relevant clinical correlates of anorexia of aging (34, 35). Selective nutritional deficits can impact the health status also in the absence of overt malnutrition. For instance, insufficient protein intake increases the risk of developing sarcopenia and is associated with morbidity and mortality (35, 36). However, more emphasis should be placed on optimal nutritional AAs balance seeking to provide primarily a sufficient EAAs supply and carefully avoiding excessive NEAAs content.

It is necessary to underline that this study was conducted on a mouse experimental model, therefore extrapolating these data on humans requires caution. Indeed, humans are not subjected to the same energetic needs as mice, and human metabolism depends also on the nutrients demands induced from environment, cognitive actions, daily activities, etc. However, interestingly, both in animals EAAs supplementation to normal diet increases life-span (5), and in humans, although EAAs enriched diets contribution to prolongation of life has never been studied yet, supplementation with EAAs to diets improves glycemic metabolism, peripheral muscle and heart performances (37, 38), and reduces infection risks in hospitalized elderly (39).

With these premises, we believe that the message that emerges from our data may be useful to reconsider some aspects of diet in humans, especially if under conditions of increased metabolic demand. Further studies in human should be performed to evaluate the use of EAAs for promoting optimal health.

**Figure 3:**
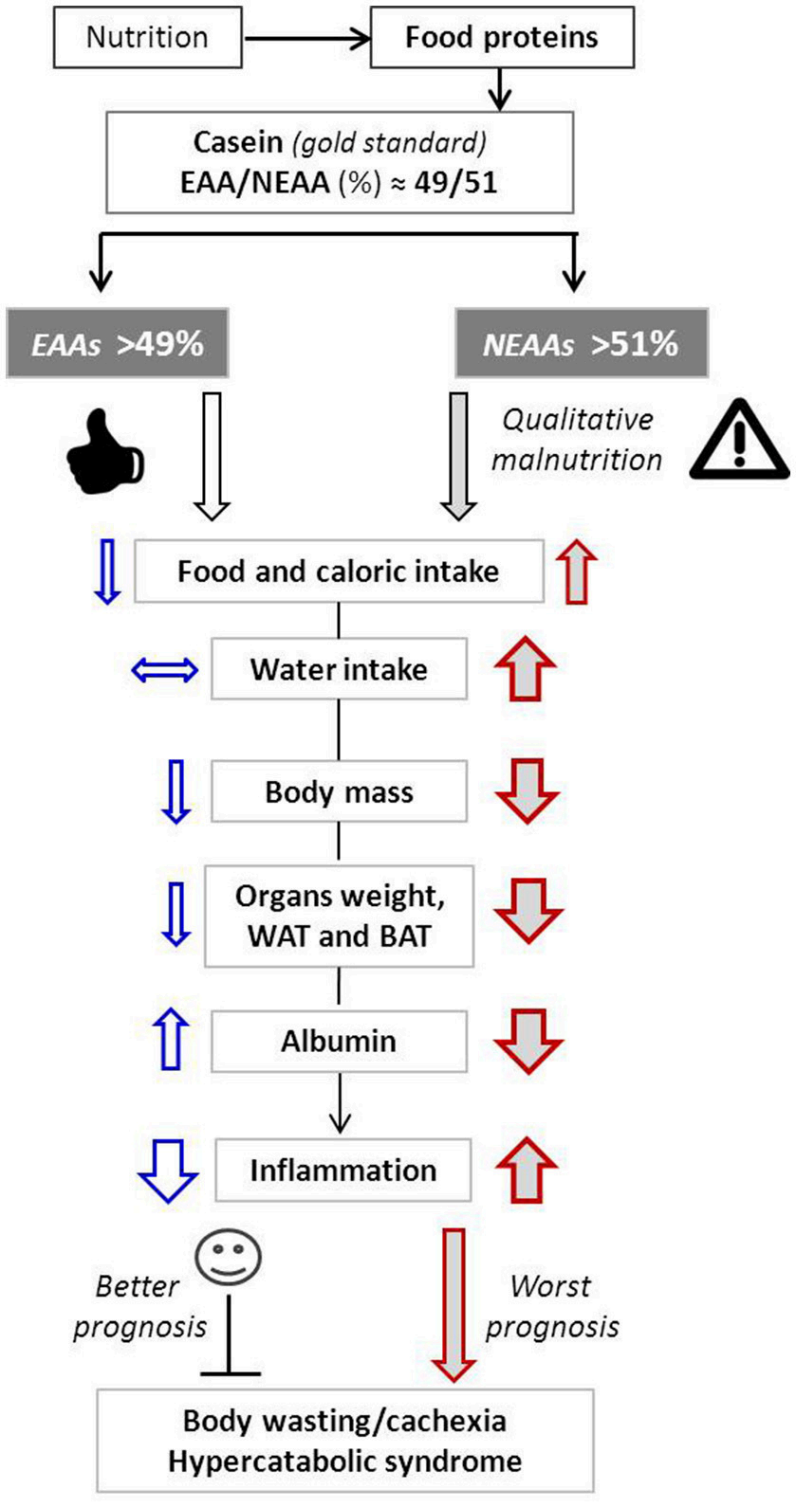
Schematic representation of the main metabolic effects induced by variation of EAA/NEAA ratios. Arrow up, increase; Arrow down, decrease; Horizontal arrow, no changes. Arrow thickness represents the magnitude of changes.

The potential events linking changes of the EAA/NEAA ratio to overall health are depicted in Figure 3.

### Study limitations

Although reporting novel findings, our study shows some limitations that need to be discussed. First of all, the study is preliminary in nature and is based on clinical and morphometric parameters, because no histopathological and molecular data are available at this stage. However, we think that these results may help understand the effects of nutrition, and/or to review certain nutritional paradigms. In addition to this, the present data could provide insights for the management of malnourished patients. Further studies are required to provide mechanistic evidence of catabolic activation induced by different dietary AAs compositions in different target organs.

## Conclusion

While tolerating excessive intake of EAAs, EAA deficiencies are deleterious in mammals. Indeed, even slightly imbalanced EAA/NEAA ratio can impact health and lifespan (40). Interestingly, loss of body weight and organ tissue wasting seem to be independent of calorie intake and may be primarily controlled by the amount of NEAAs and EAAs ingested. In our experimental conditions, malnutrition drives hypoalbuminemia and precedes and modulates inflammation. Therefore, optimizing the balance of AAs may be relevant in clinical settings related both to unwanted weight loss and the risks connected to body wasting conditions mainly in elderly people.

## Author contributions

GC, CR, and FD: Designed research; GC and CR: Conducted research; GC, EP, VF, and FD: Analyzed and interpreted data; EM, AP, and RC: Contributed to data interpretation and the drafting of the manuscript; GC, EP, and FD: Wrote the paper. All authors read and approved the final manuscript.

### Conflict of interest statement

FD is the inventor and owner of US patents: N°-US6218420 B1. Compositions based on amino-acids for preventing and treating alimentary overload in conditions of high body nitrogen requirements, without causing calcium losses. N°-US7973077 B2: Amino acid based compositions for the treatment of pathological conditions distinguished by insufficient mitochondrial function and other patents pending on different amino acid based formulations. RC and EM are partners of the SPRINTT consortium, which is partly funded by the European Federation of Pharmaceutical Industries and Associations (EFPIA). EM served as a consultant for Huron Consulting Group, Genactis, and Novartis. RC served as a consultant from Novartis and Nutricia. The other authors declare that the research was conducted in the absence of any commercial or financial relationships that could be construed as a potential conflict of interest.

## References

[1] Smerage GH. Matter and energy flows in biological and ecological systems. J Theor Biol. (1976) 57: 203–23. 10.1016/S0022-5193(76)80013-6 957653

[2] Hou Y Yin Y Wu G. Dietary essentiality of “nutritionally non-essential amino acids” for animals and humans. Exp Biol Med. (2015) 240:997–1007. 10.1177/1535370215587913 26041391 PMC4935284

[3] Corsetti G Pasini E D'Antona G Nisoli E Flati V Assanelli D. Morphometric changes induced by amino acid supplementation in skeletal and cardiac muscles of old mice. Am J Cardiol. (2008) 101:S26–34. 10.1016/j.amjcard.2008.02.078 18514623

[4] Flati V Pasini E D'Antona G Speca S Toniato E Martinotti S. Intracellular mechanisms of metabolism regulation: the role of signaling via the mammalian target of rapamycin pathway and other routes. Am J Cardiol. (2008) 101:16E−21. 10.1016/j.amjcard.2008.02.075 18514621

[5] D'Antona G Ragni M Cardile A Tedesco L Dossena M Bruttini F. Branched-chain amino acid supplementation promotes survival and supports cardiac and skeletal muscle mitochondrial biogenesis in middle-aged mice. Cell Metab. (2010) 12:362–72. 10.1016/j.cmet.2010.08.016 20889128

[6] Stacchiotti A Corsetti G Lavazza A Rezzani R Microscopic features of mitochondria rejuvenation by amino acids, in: Antonio Méndez-Vilas editor, Current microscopy contributions to advances in science and technology. Formatex Research Center (2012) 1:286–94.

[7] Corsetti G Stacchiotti A Tedesco L D'Antona G Pasini E Dioguardi FS. Essential amino acid supplementation decreases liver damage induced by chronic ethanol consumption in rats. Int J Immunopathol Pharmacol. (2011) 24:611–19. 10.1177/039463201102400307 21978693

[8] Tedesco L Corsetti G Ruocco C Ragni M Rossi F Carruba MO. A specific amino acid formula prevents alcoholic liver disease in rodents. Am J Physiol Gastrointest Liver Physiol. (2018). [Epub ahead of print]. 10.1152/ajpgi.00231.2017 29368944

[9] Corsetti G D'Antona G Ruocco C Stacchiotti A Romano C Tedesco L Dietary supplementation with essential amino acids boots the beneficial effects of rosuvastatin on mouse kidney. Amino Acids (2014) 46:2189–203. 10.1007/s00726-014-1772-5 24923264 PMC4133027

[10] Corsetti G Romano C Pasini E Marzetti E Calvani R Picca A. Diet enrichment with a specific essential free amino acid mixture improves healing of undressed wounds in aged rats. Exp Geront. (2017) 96:138-45. 10.1016/j.exger.2017.06.020 28669821

[11] Bonfili L Cecarini V Cuccioloni M Angeletti M Flati V Corsetti G. Essential amino acid mixtures drive cancer cells to apoptosis through proteasome inhibition and autophagy activation. FEBS J. (2017) 284:1726–37. 10.1111/febs.14081 28391610

[12] Young EM Food and Development. Abingdon: Routledge (2012).

[13] Ramarao PB Norton HW Johnson BC The amino acid composition and nutritive value of proteins. V. Amino acid requirements as a pattern for protein evaluation. J Nutr. (1964) 8:88–92. 10.1093/jn/82.1.88 14110945

[14] Dioguardi FS. Clinical use of amino acids as dietary supplement: pros and cons. J. Cachexia Sarcopenia Muscle (2011) 2:75–80. 10.1007/s13539-011-0032-8 21766052 PMC3118002

[15] Pasini E Aquilani R Dioguardi FS D'Antona G Gheorghiade M Taegtmeyer H. Hypercatabolic syndrome: molecular basis and effects of nutritional supplements with amino acids. Am J Cardiol. (2008) 101:S11–5. 10.1016/j.amjcard.2008.02.074 18514619

[16] Gietzen WD Hao S Anthony TG. Mechanisms of food intake repression in indispensable amino acid deficiency. Annu Rev Nutr. (2007) 27:63–78. 10.1146/annurev.nutr.27.061406.093726 17328672

[17] Ouellet G Malhotra R Penne EL Usvya L Levin NW Kotanko P. Neutrophil-lymphocyte ratio as a novel predictor of survival in chronic hemodialysis patients. Clin Nephrol. (2016) 85:191–8. 10.5414/CN108745 26951970

[18] Prats-Puig A Gispert-Saüch M Díaz-Roldán F Carreras-Badosa G Osiniri I Planella-Colomer M. Neutrophil-to-lymphocyte ratio: an inflammation marker related to cardiovascular risk in children. Thromb Haemost (2015) 114:727–34. 10.1160/TH15-01-0037 26224329

[19] Springer J Tschirner A Hartman K Palus S Wirth EK Busquets Ruis S. Inhibition of xanthine oxidase reduces wasting and improves outcome in a rat model of cancer cachexia. Int J Cancer (2012) 131:2187–96. 10.1002/ijc.27494 22336965

[20] Potier M Darcela N Tome D. Protein, amino acids and the control of food intake. Curr Opin Clin Nutr Metab Care (2009) 12:54–8. 10.1097/MCO.0b013e32831b9e01 19057188

[21] Osborne TM Mendel LB Nutritive properties of proteins of the maize kernel. J Biol Chem. (1914) 18:1–16.

[22] Cheng Y Meng Q Wang C Li H Huang Z Chen S. Leucine deprivation decreases fat mass by stimulation of lipolysis in white adipose tissue and upregulation of uncoupling protein 1 (UCP1) in brown adipose tissue. Diabetes (2010) 59:17–25. 10.2337/db09-0929 19833890 PMC2797918

[23] Mitrache C Passweg JR Libura J Petrikkos L Seiler WO Gratwohl A. Anemia: an indicator for malnutrition in the elderly. Ann Hematol. (2001) 80:295–8. 10.1007/s002770100287 11446733

[24] Ou SM Chen YT Hung SC Shih CJ Lin CH Chiang CK Taiwan Geriatric Kidney Disease (TGKD) Research Group: association of estimated glomerular filtration rate with all-cause and cardiovascular mortality: the role of malnutrition-inflammation-cachexia syndrome. J Cachexia Sarcopenia Muscle (2016) 7:144–51. 10.1002/jcsm.12053 27493868 PMC4864176

[25] Evans WJ Morley JE Argilés J Bales C Baracos V Guttridge D. Cachexia: a new definition. Clin Nutr. (2008) 27:793–9. 10.1016/j.clnu.2008.06.013 18718696

[26] Baracos VE DeVivo C Hoyle DH Goldberg AL. Activation of the ATP–ubiquitin–proteasome pathway in skeletal muscle of cachectic rats bearing a hepatoma. Am J Physiol. (1995) 268:E996–1006. 7539218 10.1152/ajpendo.1995.268.5.E996

[27] Gietzen DW Magrum LJ. Molecular mechanisms in the brain involved in the anorexia of branched-chain amino acid deficiency. J Nutr (2001) 131:851S−5. 10.1093/jn/131.3.851S 11238773

[28] Leib DE Knight ZA. Re-examination of dietary amino acid sensing reveals a GCN2-independent mechanism. Cell Rep. (2015) 13:1081–9. 10.1016/j.celrep.2015.09.055 26526991 PMC4836942

[29] Gietzen DW Anthony TG Fafournoux P Maurin AC Koehnle TJ Hao S. Measuring the ability of mice to sense dietary essential amino acid deficiency: the importance of amino acid status and timing. Cell Reports (2016) 16:2049–50. 10.1016/j.celrep.2016.08.021 27558824

[30] Iwasaki K Kasahara T Sato M. Gustatory effectiveness of amino acids in mice: behavioral and neurophysiological studies. Physiol. Behav. (1985) 34:531–42. 10.1016/0031-9384(85)90045-9 4011734

[31] Layman DK. Dietary Guidelines should reflect new understandings about adult protein needs. Nutr Metab. (2009) 6:12. 10.1186/1743-7075-6-12 19284668 PMC2666737

[32] Dubos R Schaedler RW Costello R. Lasting biological effects of early environmental influences. I. Conditioning of adult size by prenatal and postnatal nutrition. J Exp Med. (1968) 127:783–99. 10.1084/jem.127.4.783 5642467 PMC2138468

[33] Landi F Picca A Calvani R Marzetti E. Anorexia of aging: assessment and management. Clin Geriatr Med. (2017) 33:315–23. 10.1016/j.cger.2017.02.004 28689565

[34] Landi F Russo A Liperoti R Tosato M Barillaro C Pahor M. Anorexia, physical function, and incident disability among the frail elderly population: results from the ilSIRENTE study. J Am Med Dir Assoc. (2010) 11:268–74. 10.1016/j.jamda.2009.12.088 20439047

[35] Landi F Liperoti R Russo A Giovannini S Tosato M Barillaro C Association of anorexia with sarcopenia in a community-dwelling elderly population: results from the “il SIRENTE” study. Eur J Nutr. (2013) 52:1261–8. 10.1007/s00394-012-0437-y 22923016

[36] Landi F Liperoti R Lattanzio F Russo A Tosato M Barillaro C. Effects of anorexia on mortality among older adults receiving home care: an observation study. J Nutr Health Aging (2012) 16:79–83. 10.1007/s12603-011-0064-y 22238005 PMC12878108

[37] Scognamiglio R Negut C Piccolotto R Dioguardi FS Tiengo A Avogaro A. Effects of oral amino acid supplementation on myocardial function in patients with type 2 diabetes mellitus. Am Heart J. (2004) 147:1106–12. 10.1016/j.ahj.2003.12.003 15199363

[38] Scherbakov N Ebner N Sandek A Meisel A Haeusler KG von Haehling S Influence of essential amino acids on muscle mass and muscle strength in patients with cerebral stroke during early rehabilitation: protocol and rationale of a randomized clinical trial (AMINO-Stroke Study). BMC Neurol. (2016) 22 16:10. 10.1186/s12883-016-0531-5 PMC4722757 26793971

[39] Aquilani R Zuccarelli GC Dioguardi FS Baiardi P Frustaglia A Rutili C. Effects of oral amino acid supplementation on long-term-care-acquired infections in elderly patients. Arch Gerontol Geriatr. (2011) 52:e123–8. 10.1016/j.archger.2010.09.005 20934757

[40] Baron H. Some effects of DL-Methionine and Glycocyamine on growth and nitrogen retention in rats. J Nutr. (1958) 64:229–39. 10.1093/jn/64.2.229 13526006

